# *E. coli* Nickel‐Iron Hydrogenase 1 Catalyses Non‐native Reduction of Flavins: Demonstration for Alkene Hydrogenation by Old Yellow Enzyme Ene‐reductases**

**DOI:** 10.1002/anie.202101186

**Published:** 2021-05-11

**Authors:** Shiny Joseph Srinivasan, Sarah E. Cleary, Miguel A. Ramirez, Holly A. Reeve, Caroline E. Paul, Kylie A. Vincent

**Affiliations:** ^1^ Department of Chemistry University of Oxford Inorganic Chemistry Laboratory South Parks Rd Oxford OX1 3QR United Kingdom; ^2^ Department of Biotechnology Delft University of Technology Van der Maasweg 9 2629 HZ Delft The Netherlands

**Keywords:** asymmetric catalysis, biocatalysis, cofactor recycling, ene-reductase, hydrogenation

## Abstract

A new activity for the [NiFe] uptake hydrogenase 1 of Escherichia coli (Hyd1) is presented. Direct reduction of biological flavin cofactors FMN and FAD is achieved using H_2_ as a simple, completely atom‐economical reductant. The robust nature of Hyd1 is exploited for flavin reduction across a broad range of temperatures (25–70 °C) and extended reaction times. The utility of this system as a simple, easy to implement FMNH_2_ or FADH_2_ regenerating system is then demonstrated by supplying reduced flavin to Old Yellow Enzyme “ene‐reductases” to support asymmetric alkene reductions with up to 100 % conversion. Hyd1 turnover frequencies up to 20.4 min^−1^ and total turnover numbers up to 20 200 were recorded during flavin recycling.

## Graphical abstract

Robust [NiFe] hydrogenase 1 (Hyd1) from *Escherichia coli* is shown to have non‐native, H_2_‐dependent activity for FMN and FAD reduction. It is a promising recycling system for FMNH_2_ or FADH_2_ supply to flavoenzymes for chemical synthesis when coupled with an Old Yellow Enzyme ene‐reductase.

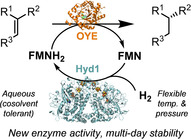

Academic and industrial fields are increasingly looking to biotechnology to make chemical manufacturing more sustainable.^[1]^ Enzymes provide many advantages: they are renewable, biodegradable, nonhazardous, and provide high selectivity. Furthermore, the once‐limited scope of known enzyme reactions is rapidly expanding, aided by enzyme engineering and ongoing discovery and characterization of new enzymatic functions.[^2^, ^3^]

Old Yellow Enzyme (OYE) ene‐reductases are gaining prominence in industrial biotechnology for catalysis of asymmetric alkene reductions. OYEs contain a tightly bound FMN prosthetic group which transfers electrons from an external reductant to an activated alkene (Supporting Information, Figure S2). Most commonly, OYEs are supplied with reducing equivalents via the expensive cofactors NADPH or NADH, and hence they are typically operated with a cofactor recycling system for the reduced nicotinamide cofactors such as glucose/glucose dehydrogenase (GDH). OYE ene‐reductases can also accept reducing equivalents from synthetic analogues of NADH,^[4]^ although work is still needed on effective recycling systems for these artificial cofactors. There are also reports[^5^, ^6^] of electron uptake from reduced flavins, FMNH_2_ or FADH_2_ (oxidized and reduced forms are shown in Scheme 1). Presumably the tightly bound prosthetic flavin in OYEs is sufficiently exposed to allow this promiscuity in terms of reductant. Supply of a catalytic quantity of oxidized FMN or FAD, together with a recycling system for reduced flavin is preferable to stoichiometric addition of FMNH_2_ or FADH_2_, both in terms of lowering cost and minimizing waste. Reduced flavins have been recycled in situ by means of photochemistry, electrochemistry or metal catalysis,^[6]^ which can suffer from biocompatibility challenges (such as mutual inactivation, mismatched ideal solvent, pH, or temperature).[^7^, ^8^] Milder biocatalytic approaches to flavin recycling are cumbersome (Supporting Information, Figure S3),[^7^, ^9^, ^10^] requiring both an NAD(P)H‐dependent reductase to produce FMNH_2_ or FADH_2_ at the expense of NAD(P)H^[11]^ and GDH/glucose for recycling the NAD(P)H.

**Scheme 1:**
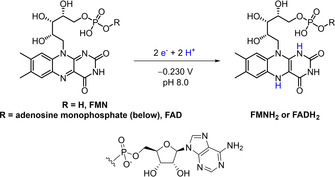
Oxidized (left) and reduced (right) FMN or FAD cofactors.

Use of H_2_ for cleaner enzymatic NAD(P)H cofactor recycling has been demonstrated.[^12^, ^13^, ^14^] The soluble hydrogenase from *Cupriavidus necator* (formerly *Ralstonia eutropha*) natively uses H_2_ to provide electrons for NAD^+^ reduction at a prosthetic flavin cofactor.^[13]^ Reduction of external flavin substrates by this enzyme under H_2_ has long been known,^[15]^ and presumably occurs at the NAD^+^ binding site. The multi‐subunit soluble hydrogenase has recently been demonstrated as a possible recycling system for reduced flavin,^[16]^ but the enzyme is complex to express and lacks stability.[^17^, ^18^]

This inspired us to test whether a simple hydrogenase (Figure 1) could be suitable for H_2_‐driven flavin reduction. The thermodynamic potential for the H^+^/H_2_ couple (−0.472 V, pH 8) relative to the flavin potential (−0.230 V, pH 8),^[19]^ makes reduction of flavin by H_2_ thermodynamically favorable. We selected *E. coli* [NiFe]‐hydrogenase (Hyd1), which is a good H_2_ oxidizer[^20^, ^21^] and well‐characterized in terms of X‐ray crystal structures[^22^, ^23^] and spectroscopy.[^21^, ^24^] Hyd1 is natively expressed in *E. coli* and, unlike many hydrogenases,^[25]^ it is O_2_‐tolerant^[21]^ and active over a wide pH range.^[26]^ Like other uptake hydrogenases, the basic unit of Hyd1 is a heterodimer of the large subunit (HyaB) housing the [NiFe] active site, and the small subunit (HyaA) housing the iron‐sulfur cluster electron transfer relay. Natively, Hyd1 exists as a homodimer, (HyaAB)_2_ and is coupled to a cytochrome electron acceptor. Our isolated enzyme comprises predominantly the dimeric HyaAB^[27]^ and our preparation lacks the cytochrome (Supporting Information, Figure S1).

**Figure 1:**
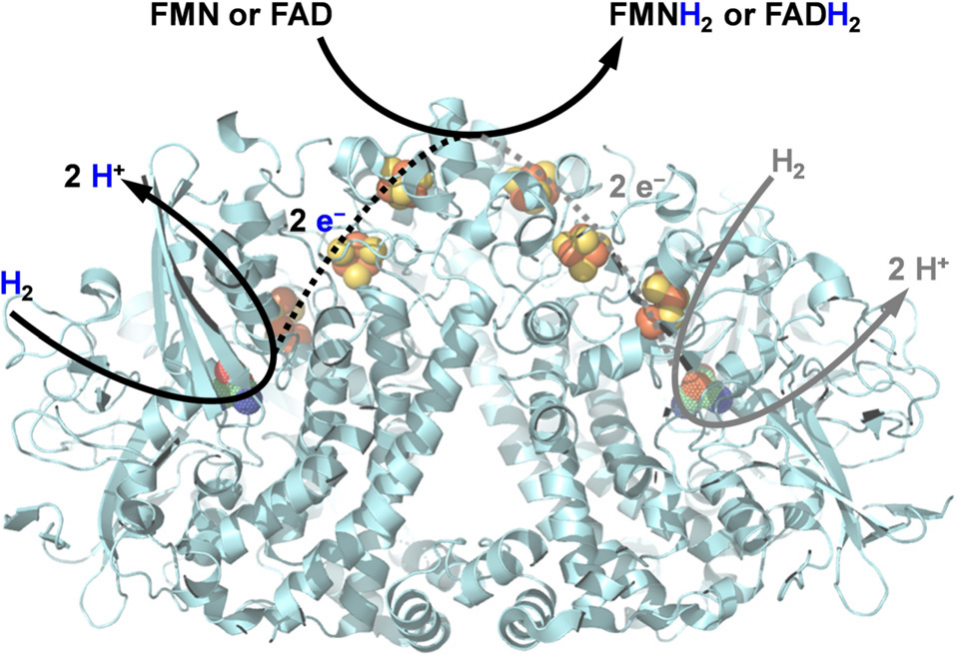
Flavin reduction by Hyd1. H_2_ oxidation at the [NiFe] active site (green, red, blue) provides 2 electrons that are transferred to the protein surface via FeS clusters (yellow, orange). The figure, showing the homodimer of HyaAB units, was prepared using PyMOL 2.3.4 (PDB: 6FPW).

The H_2_ oxidation activity of Hyd1 is typically measured using the artificial electron acceptor benzyl viologen in colourimetric assays.^[26]^ Electrons from H_2_ oxidation at the [NiFe] active site (Figure 1) are relayed through FeS clusters where, evidence suggests, benzyl viologen reduction occurs, rather than directly at the [NiFe] active site.^[28]^ The fact that electron transfer from hydrogenases to electrodes is also well‐established[^21^, ^25^] encouraged us to explore scope for other non‐natural electron transfer reactions of robust Hyd1 from *E. coli*. We demonstrate that both FMN and FAD can accept electrons from H_2_ oxidation by Hyd1 to generate FMNH_2_ and FADH_2_ respectively, and show that Hyd1 can be used as an effective FMNH_2_ regeneration system to support asymmetric alkene reduction by three OYE‐type ene‐reductases.

Figure 2 shows the results of in situ UV/Vis spectrophotometric assays to explore H_2_‐driven FMN and FAD reduction by Hyd1 (produced and isolated in accord with the Supporting Information, Methods Section S1.2; reaction follows General Procedure A). The flavin moiety of FMN gives *λ*_max_ at 445 nm and FAD at 450 nm, both of which bleach upon two‐electron reduction[^29^, ^30^] (Figure 2 A,B; see the Supporting Information, Figure S6 for spectra of fully reduced FMN). The decrease in oxidized flavin concentration over time was used to calculate initial enzyme activity (Figure 2 C,D). Control experiments indicated that omission of Hyd1 or H_2_ led to negligible flavin reduction (Supporting Information, Figures S7–S9).

**Figure 2:**
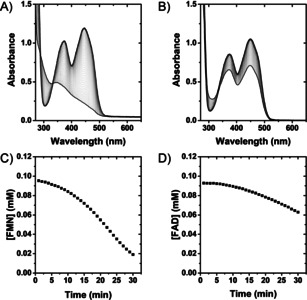
Activity assay for H_2_‐driven Hyd1 reduction of flavin measured by in situ UV/Vis spectroscopy. A) Hyd1 reducing FMN. B) Hyd1 reducing FAD. C) Calculated [FMN] based on *λ*_max_=445 nm (*ϵ*=12.50 mM^−1^ cm^−1^). D) Calculated [FAD] based on *λ*_max_=450 nm (*ϵ*=11.30 mM^−1^ cm^−1^). Reaction conditions: General Procedure A in Tris‐HCl buffer (50 mM, pH 8.0, 25 °C).

Upon addition of Hyd1, a lag phase was observed during FMN and FAD reduction, which is attributed to the well‐characterized H_2_‐dependent activation phase for aerobically purified Hyd1.^[21]^ Later experiments (when indicated) used Hyd1 that was first activated under a H_2_ atmosphere.^[31]^ The lag phase was followed by a decrease in absorbance consistent with FMNH_2_/FADH_2_ formation, and clear isosbestic points at 330 nm corroborate a lack of side products. Specific initial activities for FMN and FAD reduction (76 and 32 nmol min^−1^ mg^−1^ Hyd1, respectively) were determined during the linear reaction phase. The higher activity for reduction of FMN compared with FAD cannot be attributed to thermodynamic driving force since both cofactors have similar reduction potentials,^[19]^ but could relate to the cofactors’ ability to interact at the protein surface.

Hyd1 is known to be robust which inspired us to test H_2_‐driven flavin reduction activity at different temperatures (25–70 °C, General Procedure A). Figure 3 shows the conversions from reactions performed at different temperatures after 30 minutes relative to a standard reaction performed at 25 °C. This standard temperature and stop time were selected to leave room for improvement in conversions of FMN and FAD at the higher temperatures. Reactions at 25–50 °C using FMN were performed twice, and the corresponding bars indicate the average relative conversion with the range of results represented with error bars (±3–12 %). This level of reproducibility is likely to extend to FAD owing to an identical reaction set up. Results for FMN and FAD may not be directly comparable due to different purity levels of the cofactors which were obtained from different suppliers. Conversion of FMN and FAD to the reduced forms after 30 min reaction time increased with temperature (Figure 3), suggesting that Hyd1 is likely to open new doors to cofactor recycling for flavoenzymes with optimal activity at higher temperatures.

**Figure 3:**
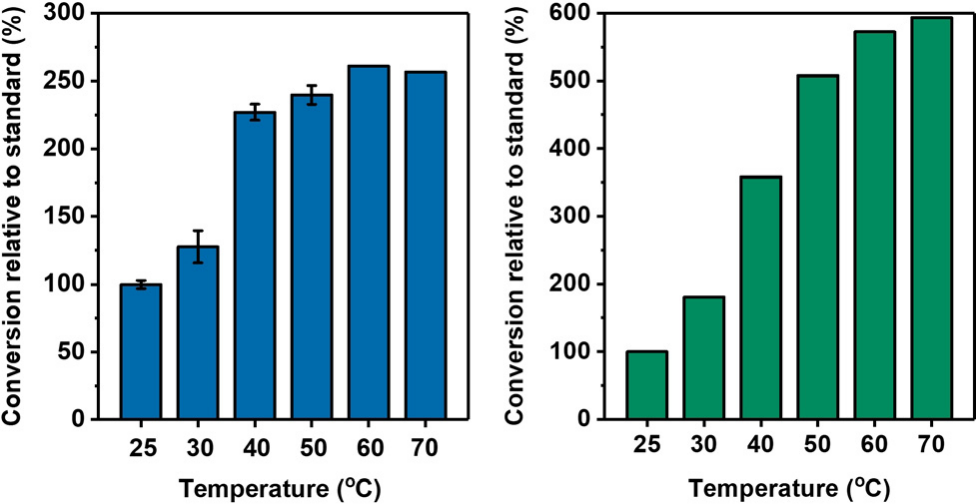
Hyd1‐catalysed flavin reduction at different temperatures (left: FMN; right: FAD). Conversion relative to standard=$\frac{\mathrm{Conversion}\;\mathrm{at}\;\mathrm{temp}}{\mathrm{Conversion}\;\mathrm{at}\;25{\;}^{\circ }C\;}\;\times 100\%$ . The FMN 25–50 °C bars represent the average of relative conversions calculated from duplicate experiments, with the range represented as error bars. Reaction conditions: General Procedure A (Supporting Information) in phosphate buffer (50 mM, pH 8.0). Conversion was calculated after 30 min using UV‐visible spectroscopy.

To demonstrate the utility of Hyd1 in biotechnologically‐relevant flavin recycling, we first coupled Hyd1‐catalysed flavin reduction with the OYE‐type ene‐reductase from *Thermus scotoductus, Ts*OYE,[^32^, ^33^] to catalyze enantioselective reduction of ketoisophorone (**1**) to (*R*)‐levodione (**2**, Table 1). Reactions were conducted according to General Procedure B (Supporting Information) and monitored using chiral‐phase GC‐FID after extraction of the reaction mixture into ethyl acetate (Supporting Information, Figure S13). Enantiomeric excess (*ee*) was always >99 % at the first time point but decreased to 86–92 % from slow racemization under alkaline conditions as previously reported.^[34]^ Control experiments confirmed good reproducibility (4.4 % standard deviation) and that each component is required for conversion (Supporting Information, Tables S1,S2).

**Table 1:** H_2_‐driven enzymatic reduction of **1** under various conditions.^[a]^ 
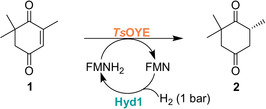

Entry	[**1**] (mM)	[FMN] (mM)	Conv. to [%]^[b]^	Hyd1 TOF [min^−1^]^[c]^	Hyd1 TTN^[d]^	FMN TN^[d]^
1	2	0.5	100	20.4	2100	4
2	2	0.1	100	7.8	2100	20
3	5	0.1	95 {100}	4.8	5200	50
4	10	0.1	62 {97}	5.4	10 200	97
5	20	0.1	24 {37}	5.4	7800	74
6^[e]^	20	0.1	{44}	8.4	9300	88
7^[f]^	10	0.1	{94}	9.6	9900	94
8^[g]^	20 then 24.2^[h]^	0.1	{29} then >99^[i]^	3.0	20 200^[i]^	240^[i]^

[a] Reaction conditions: In accord with General procedure B using 57 μg Hyd1, 72 μg *Ts*OYE in Tris‐HCl (50 mM, pH 8), 1 vol % DMSO at room temperature (20 °C–22 °C). [b] GC conversion to **2** at 15 h {and 24 h}. [c] Hyd 1 turnover frequency (mol **2** per mol Hyd1 per min) was calculated after 60 minutes. [d] Hyd1 total turnover number (mol **2** per mol Hyd1) and FMN turnover number (mol **2** per mol FMN) were determined at the end of the reaction. [e] 4 bar H_2_. [f] 35 °C; some evaporation of **1** and **2** was observed from GC‐FID. [g] 71 μg Hyd1 was used. [h] Reaction was fed with additional 72 μg *Ts*OYE and 4.2 mM **1** at 66 h and 71 h, respectively. [i] Conversion, Hyd 1 TTN and FMN TN were determined at 134 hours, additional time point data in the Supporting Information, Figure S12.

The highest Hyd1 turnover frequency (TOF, 20.4 min^−1^) and quantitative conversion after 15 h were achieved with 0.5 mM FMN and 2 mM **1** at room temperature (entry 1, Table 1).

When 0.1 mM FMN was used with varying [**1**] (entries 2–5), a Hyd1 total turnover number (TTN) of up to 10 200 and 97 FMN turnovers (TN) were achieved after 24 h. This is comparable to the FMN TN reported for formate‐driven Rh‐catalyzed FMNH_2_ recycling, however background, non‐enantioselective reduction of **1** by [Cp*Rh(bpy)H]^+^ meant a careful balance of catalysts was required in that case.^[32]^ This was not an appreciable issue with our biocatalytic system (Supporting Information, Table S2). Increasing H_2_ pressure to 4 bar boosted conversion and Hyd1 TOF from 5.4 min^−1^ to 8.4 min^−1^, likely due to improved H_2_ availability (entries 5,6).

Like Hyd1, *Ts*OYE has enhanced activity at elevated temperatures,^[33]^ therefore entry 4 was replicated at 35 °C (see entry 7). Hyd1 TOF nearly doubled to 9.6 min^−1^ and 94 % conversion was achieved after 24 h, however GC‐FID showed that some of **1** and **2** likely evaporated.

To test stability over time, entry 5 was replicated using 71 μg Hyd1, and as the reaction neared full conversion an additional 72 μg *Ts*OYE then 4.2 mM **1** was added (66 h and 71 h, respectively, see entry 8). Though the reaction likely still had active enzymes (Supporting Information, Figure S12), the reaction was stopped for analysis at 134 h (5.5 days) after which Hyd1 TTN reached 20 200 and FMN TN 240. This represents an improvement in stability over *R. eutropha* SH (TTN 8400) for flavin recycling with *Ts*OYE.^[16]^ The 20 200 TTN is of an appropriate order of magnitude for use as a catalyst in the pharmaceutical and fine chemicals industries,^[35]^ approaches values measured from commercial grade enzymatic processes,^[36]^ and there remains room for further optimization to that end. The demonstrated continuous Hyd1 stability over time (Supporting Information, Figure S12) is an important performance benchmark for potential commercial applications, particularly in flow.^[37]^ Furthermore, this application is likely to extend to *Ts*OYE variants, which have demonstrated broad substrate acceptance, are robust in harsh conditions, and can switch enantioselectivity.^[38]^

We extended this system to two commercially available ene‐reductases, ENE‐103 and ENE‐107 (Johnson Matthey), which are typically sold as a kit with GDH and formate dehydrogenase for NAD(P)H recycling. The alkene reductions demonstrated were dimethyl itaconate (**3**) reduction to dimethyl (*R*)‐methyl succinate (**4**) by ENE‐103 and 4‐phenyl‐3‐buten‐2‐one (**5**) reduction to 4‐phenyl‐2‐butanone (**6**) by ENE‐107 (Table 2), using the same protocols established for *Ts*OYE. Control experiments to show that each component is required for substrate conversion are summarized in the Supporting Information, Tables S3,S4.

**Table 2:** H_2_‐driven enzymatic alkene reductions using commercial ene‐reductases.^[a]^ 
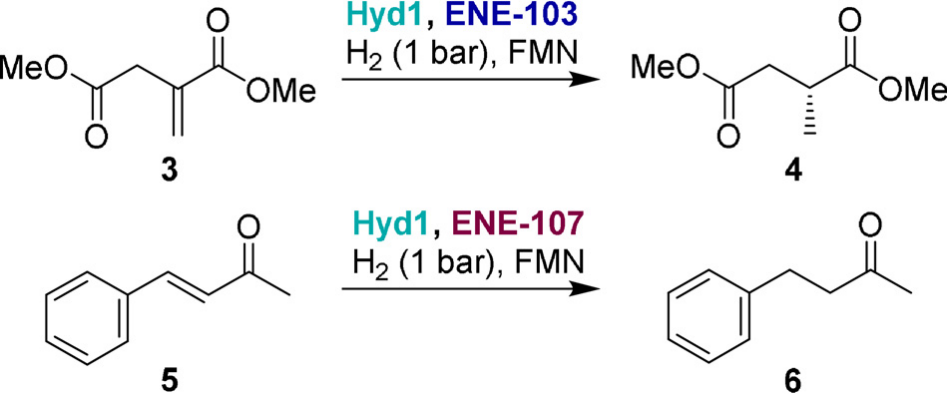

Entry	Substrate	[FMN] (mM)	Ene‐reductase	*t* [h]	Conv. [%]^[b]^	*ee* [%]
1	**3**	0.1	ENE‐103	42	81	>99
2	**3**	0.5	ENE‐103	42	98	>99
3^[c]^	**5**	0.1	ENE‐107	24	20±1	n.a.^[d]^
4^[c]^	**5**	0.5	ENE‐107	24	33±3	n.a.^[d]^
5	**5**	0.1	ENE‐107	40	35	n.a.^[d]^
6	**5**	0.5	ENE‐107	40	100	n.a.^[d]^

[a] Reaction conditions: In accord with General procedure B using 142 μg Hyd1, 3 mg ene‐reductase and 5 mM substrate in Tris‐HCl (50 mM, pH 8), 1 vol% DMSO at room temperature (20 °C–30 °C). [b] GC conversions to **4** or **6**. [c] Entries 3 and 4 were performed in triplicate and are shown ±1 standard deviation, and were separate experiments from entries 5 and 6. [d] Not applicable.

With ENE‐103, enantioselective (>99 % *ee*) reduction to (*R*)‐**4** improved from 81 % to 98 % conversion as FMN concentration was increased from 0.1 mM to 0.5 mM (entries 1,2). Conversion of **5** to **6** using ENE‐107 was drastically improved when FMN concentration increased from 0.1 mM to 0.5 mM (compare entries 3 and 4, and entries 5 and 6), increasing from 35 % to 100 % conversion in the 40 hour experiment. These results highlight the straightforward application of different ene‐reductases with Hyd1‐catalysed flavin recycling, suggesting that this simplified H_2_‐driven system could be valuable in applications that require low waste, high catalyst stability and temperature tolerance.

Our work has shown a clean, atom‐efficient way of driving commercial ene‐reductase enzymes with flavin recycling in place of nicotinamide cofactor recycling. Further modifications to Hyd1, which is tolerant of mutagenesis,[^23^, ^31^] might enhance its non‐native flavin reduction activity. Other promising synthetically interesting flavin‐dependent enzymes, including halogenases (chlorination, bromination, iodination)^[7]^ and flavoprotein monooxygenases (epoxidation, hydroxylation, Baeyer–Villiger oxidation)[^39^, ^40^] are currently under‐utilized in industrial biotechnology, perhaps due to the lack of available simplified flavin recycling systems. This proof‐of‐concept work shows that the robust Hyd1, tolerant to a range of conditions, is a promising catalyst to develop for clean flavin recycling in biotechnology.

## Conflict of interest

The authors declare no conflict of interest.

## Supporting information

As a service to our authors and readers, this journal provides supporting information supplied by the authors. Such materials are peer reviewed and may be re‐organized for online delivery, but are not copy‐edited or typeset. Technical support issues arising from supporting information (other than missing files) should be addressed to the authors.

Supplementary
