# Supplementary material for: E. coli Nickel‐Iron Hydrogenase 1 Catalyses Non‐native Reduction of Flavins: Demonstration for Alkene Hydrogenation by Old Yellow Enzyme Ene‐reductases
Source: Angew Chem Int Ed Engl. 2021 May 11;60(25):13824–8. doi: 10.1002/anie.202101186 (PMC8252551; doi:10.1002/anie.202101186)
Supplement: Supplementary file 1 — Supplementary [file ANIE-60-13824-s001.pdf]

Angewandte Chemie
Eine Zeitschrift der Gesellschaft Deutscher Chemiker

Supporting Information

E. coli Nickel-Iron Hydrogenase 1 Catalyses Non-native Reduction of Flavins: Demonstration for Alkene Hydrogenation by Old Yellow Enzyme Ene-reductases**

*Shiny Joseph Srinivasan, Sarah E. Cleary, Miguel A. Ramirez, Holly A. Reeve, Caroline E. Paul, and Kylie A. Vincent**

anie_202101186_sm_miscellaneous_information.pdf

Table of contents

S1. Reagents 1
S1.1. General reagents 1
S1.2. Enzymes 2
S1.2.1. *E. coli* hydrogenase 1 2
S1.2.2. *Thermus scotoductus* ene-reductase, *Ts*OYE 2
S1.2.3. Commercial ene-reductases (ENE-103 and ENE-107) 3
S2. Analytical tools 3
S2.1. UV-visible spectroscopy to monitor flavin reduction 3
S2.2. Chiral phase GC-FID to monitor alkene reductions 3
S2.2.1. Reduction of Ketoisophorone (1) and 4-phenyl-3-buten-2-one (5) 3
S2.2.2. Reduction of Dimethyl itaconate (3) 4
S3. Experimental procedures 4
S3.1. General Procedure A (Flavin reduction) 4
S3.2. General Procedure B (Alkene reduction) 5
S3.3. General Procedure C (Preparing samples for chiral GC analysis) 5
S4. Supplementary data and results 5
S4.1. Kinetic studies to determine the K_m (Michaelis-Menten constant) of Hyd1 for FAD . 8
S4.2. Complete reduction of FMN to $FMNH_2$ 9
S4.3. Control experiments for Hyd1 catalysed flavin reduction 9
S4.4. Reduction of FMN using Hyd1 enzyme that contains or lacks the cytochrome 11
S4.6. Reproducibility of the reactions 12
S4.7. Control experiments for H_2-driven alkene reduction 13
S4.8. Exemplary chiral-phase GC-FID spectra of H_2-driven alkene reductions 14
S5. References 17

S1. Reagents

S1.1. General reagents

Buffer salts (Sigma-Aldrich), FAD (disodium salt, ≥98%, Cayman Chemical Company), and FMN (monosodium salt dihydrate, Applichem Panreac) were all used as received. Ketoisophorone (**1**, 2,6,6-trimethyl-2-cyclohexene-1,4-dione, ≥98%), dimethyl (*R*)-(+)-methyl succinate, ((*R*)-**4** (99%)), *rac*-**4** (98%), 4-phenyl-3-buten-2-one (**5,** 99%) and 4-phenyl-2-butanone (**6,** 98%) were purchased from Sigma-Aldrich. Dimethyl itaconate (**3**, 98%) was purchased from Fluorochem. (6*R*)-Levodione ((*R*)**-2**) was obtained by Baker's yeast fermentation and was a gift from Dr. Adrie Straathof from the Delft University of Technology. GC standard *rac*-**2** was prepared following literature procedure[1]. All aqueous solutions were prepared with deoxygenated MilliQ water (Millipore, 18 MΩcm).

S1.2. Enzymes

S1.2.1. *E. coli* hydrogenase 1

The hydrogenase (*E. coli* hydrogenase 1, Hyd1) was produced by homologous over-expression of the genes encoding the structural subunits of the enzyme and key maturases. After Hyd1 overexpression under anaerobic bacterial growth, the enzyme was isolated following published protocols (described in the caption to **Figure S1**).[2]

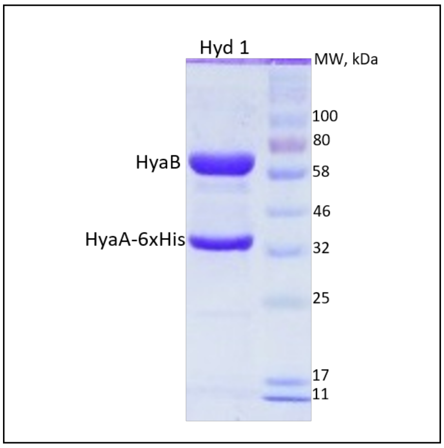

Figure S1. SDS-PAGE analysis of purified Hyd1. Typical protein composition and purity in the Hyd1 samples used. After overexpression, the Hyd1 enzyme was purified by Metal Affinity Chromatography in a Nickel-NTA prepacked column. After elution with imidazole buffer, the protein was further purified by Size Exclusion Chromatography using a HiLoad 16/600 Superdex 200 pg gel-filtration column (GE Healthcare, UK). Subsequently, the enzyme was buffer-exchanged into Tris HCl buffer (20 mM Tris-HCl pH 7.2, 350 mM NaCl, 0.02% Triton X, 1 mM DTT), concentrated and stored at −80 °C.

S1.2.2. *Thermus scotoductus* ene-reductase, *Ts*OYE

The *Thermus scotoductus* ene-reductase of the Old Yellow Enzyme family (*Ts*OYE) was produced by heterologous expression in *E. coli*, as described previously.[3,4] In brief: the gene encoding for *Ts*OYE was previously cloned in a pET-22b(+) vector, transformed into *E. coli* BL21(DE3) competent cells, which were spread on Lysogeny Broth (LB) agar plates containing ampicillin (100 µg/mL) and left to grow at 37 °C overnight. A pre-culture was started in LB medium supplemented with ampicillin (100 µg/mL) by picking one isolated colony, and shaken at 37 °C overnight. After inoculation of 1 L Terrific Broth (TB) medium (supplemented with 100 µg/mL ampicillin) with 10 mL of pre-culture, the 5-L flask was shaken in an incubator at 37 °C until the optical density at 600 nm $OD_{600\ nm}$ reached an absorbance of 0.6, then was cooled to 30 °C. Expression was induced by addition of isopropyl β-D-1-thiogalactopyranoside (IPTG, 0.5 mM). After 20 h at 30 °C, cells were harvested by centrifugation at 17,500 x *g* for 20 min at 4 °C, resuspended and washed with MOPS-NaOH buffer (20 mM, pH 7.0) and centrifuged again. The cell pellet was frozen at -80 °C overnight. The cell pellet was then thawed and resuspended in the same buffer, with DNaseI, $MgCl_2$ and a cOmplete™ EDTA-free protease inhibitor cocktail tablet, to be passed through a Multi Shot Cell Disruption System once, and centrifuged at 17,500 x *g* for 20 min at 4 °C. The resulting supernatant was heated at 70 °C for 90 min, and centrifuged at 9,500 x *g* for 30 min at 4 °C. The resulting yellow supernatant was analysed by UV-visible spectroscopy (Cary 60 spectrophotometer) to determine flavin content

(in a UV cuvette, by denaturing the enzyme sample with 0.2% sodium dodecyl sulfate, SDS) and the Bicinchoninic acid (BCA) assay was used to determine overall protein content. Flavin mononucleotide (FMN) was supplemented based on these measurements and the enzyme solution was left on ice for 30 min. The enzyme solution was then passed through a desalting column (PD-10) to remove excess FMN with MOPS-NaOH buffer (20 mM, pH 7.0), and further concentrated in an Amicon ultra centrifugal filter with a cut-off of 10 kDa, also ensuring the removal of any excess FMN. The resulting *Ts*OYE was observed to be > 95% pure (by sodium dodecyl sulfate-polyacrylamide gel electrophoresis, SDS-PAGE), frozen with liquid nitrogen and stored at −20 °C (−80 °C for long-term) as a 200 µM solution in MOPS-NaOH buffer (20 mM, pH 7.0).

S1.2.3. Commercial ene-reductases (ENE-103 and ENE-107)

Commercial samples of ene-reductases were provided by Johnson Matthey in the lyophilised forms and used without further purification.

S2. Analytical tools

S2.1. UV-visible spectroscopy to monitor flavin reduction

UV-visible spectra were recorded by a Cary 60 spectrophotometer with a cell holder (Agilent) and a Peltier accessory for temperature control using a quartz cuvette (path length 1 cm, cell volume 1 mL, Hellma). The indicated buffer was used to take a baseline scan. In some of the experiments, there was a uniform shift of the baseline across the entire spectral region (200–800 nm), which was corrected for during data processing. The concentration of FMN was directly calculated based on the absorbance at λ = 445 nm (ε = 12.50 mM^{-1} cm^{-1}) and FAD based on the absorbance at λ = 450 nm (ε = 11.30 mM^{-1} cm^{-1}). During the linear phase of the reaction, the decrease in [oxidised flavin] over time was determined in order to calculate the hydrogenase activity (mM min^{-1}).[5]

S2.2. Chiral phase GC-FID to monitor alkene reductions

S2.2.1. Reduction of Ketoisophorone (1) and 4-phenyl-3-buten-2-one (5)

Instrument: ThermoScientific Trace 1310 GC

Column: CP-Chirasil-Dex CB (Agilent), 25 m length, 0.25 mm diameter, 0.25 µm (film thickness), fitted with a guard of 10 m undeactivated fused silica of the same diameter

Carrier: He (CP grade), 170 kPa (constant pressure)

Inlet temperature: 200 °C

Injection conditions: Splitless with split flow 60 mL/min, splitless time 0.8 min, purge 5 mL/min. Injection volume = 0.1 µL.

Detection: FID (H_2 = 35 mL/min, air = 350 mL/min, makeup N_2 = 40 mL/min, temp = 200 °C)

Oven heating profile:

Time (minutes)	Temperature
0 → 5	Hold at 70 °C
5 → 30	Ramp to 120 °C at 2 °C/min
30 → 36	Ramp to 180 °C at 10 °C/min
36 → 45	Hold at 180 °C for 5 minutes

Compound retention times (reduction of **1***)*:

Time (minutes)	Compound
12.27	Ketoisophorone (**1**)
12.68	(*R*)-Levodione (**2**)
12.80	(*S*)-Levodione (**2**)

Compound retention times (reduction of **5***)*:

Time (minutes)	Compound
14.48	4-Phenyl-3-buten-2-one (**5**)
12.86	4-Phenyl-2-butanone (**6**)

S2.2.2. Reduction of Dimethyl itaconate (3)

Instrument: ThermoFinnigan Trace GC
Column: Cyclosil-B (Agilent), 30 m length, 0.25 mm diameter, 0.25 μm (film thickness)
Carrier: He (CP grade), 100 kPa (constant pressure)
Inlet temperature: 220 °C
Injection volume: 2 μL
Detection: FID (H_2 = 35 mL/min, air = 350 mL/min, N_2 = 30 mL/min, temp = 250 °C
Oven heating profile:

Time (minutes)	Temperature
0 → 160	Hold at 70 °C
160 → 170	Ramp to 180 °C at 20 °C/min

Compound retention times:

Time (minutes)	Compound
83.67	Dimethyl itaconate (**3**)
58.50	(*R*)-Dimethyl methyl succinate (**4**)
60.76	(*S*)-Dimethyl methyl succinate (**4**)

S3. Experimental procedures

All experiments were carried out in a glovebox (Glove Box Technology Ltd) under a protective N_2 atmosphere (O_2 < 3 ppm). Stock solutions of FAD and FMN were prepared using deoxygenated buffer. Different concentrations of stock solutions of **1, 3** and **5** were prepared in DMSO such that DMSO was 1 vol% in the final reaction mixture.

S3.1. General Procedure A (Flavin reduction)

The indicated volume of Tris-HCl buffer (50 mM, pH 8.0) or phosphate buffer (50 mM, pH 8.0) was added to a UV-visible quartz cuvette, which was placed in the cell holder and allowed to warm to the indicated temperature (pre-set on the Peltier accessory) for 5 min. A baseline was recorded using the UV-visible spectrophotometer (see S2.1). A solution of 0.1 mM flavin (unless otherwise noted) in the designated buffer was next prepared in the cuvette, which was then capped with a rubber septum that was pierced with two needles to provide a gas inlet and outlet. An H_2-line was then connected and H_2 was bubbled through the flavin solution via the inlet needle for 10 minutes. The needle was then moved up to the headspace through which a continuous H_2 flow was supplied. About 0.4 mL of the flavin solution was then used to transfer a designated quantity of Hyd1 into the cuvette using a syringe and needle, and the needle and

syringe rinsed by drawing solution in and out of the cuvette. The assay was carried out by taking one scan (200–800 nm) every 30 seconds over 30 minutes.

S3.2. General Procedure B (Alkene reduction)

Using a syringe and needle, 600 µL of H_2-saturated Tris-HCl buffer (50 mM, pH 8.0, 25 °C) was transferred to a centrifuge tube (Eppendorf, 1.5 mL) that contained the required quantities of FMN and **1** or **3** or **5** in DMSO (1 vol% DMSO in total reaction mixture). A portion of this solution (approx. 0.2 mL) was used to transfer the designated quantity of Hyd1 (activated under H_2 for 3–15 h) and *Ts*OYE (72 µg) or ene reductase (3 mg) into the reaction tube in sequence *via* a needle and syringe. The lid of the centrifuge tube was pierced once with a needle, capped, and placed in a Büchi Tinyclave pressure vessel which was then charged to the designated pressure of H_2. The pressure vessel was then removed from the glovebox and wrapped in aluminum foil to exclude light in order to prevent photodecomposition of the FMN, flavoenzyme, or both.[6] The vessel was placed on a Stuart® mini see-saw rocker set to 30 oscillations/min. The extent of conversion and enantiomeric excess (%*ee*) of the products was determined by chiral GC-FID (General Procedure C).

S3.3. General Procedure C (Preparing samples for chiral GC analysis)

Aliquots (15 or 25 µL) of reaction mixture were measured and taken for analysis at the indicated time, extracted into 200 µL EtOAc, then 150 µL of the EtOAc layer was removed, dried over Na_2SO_4 and 75 µL of the solution was taken for GC analysis (see S4.7).

S4. Supplementary data and results

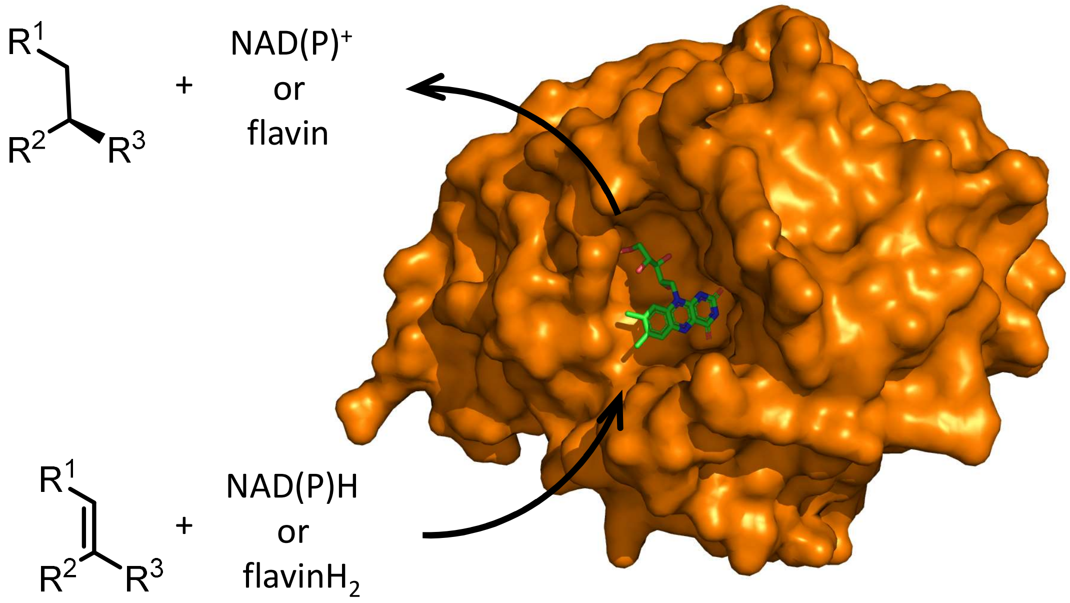

Figure S2. Alkene reduction by OYE-type ene-reductases (orange). Electrons from an external reductant (e.g. NAD(P)H or reduced flavin 'flavinH_2') reduce the tightly bound, though sufficiently exposed, prosthetic FMN (green, blue, red) which goes on to reduce an activated alkene substrate. This figure shows the monomer of *Ts*OYE, prepared using PyMOL™ 2.3.4 (PDB: 3HF3).

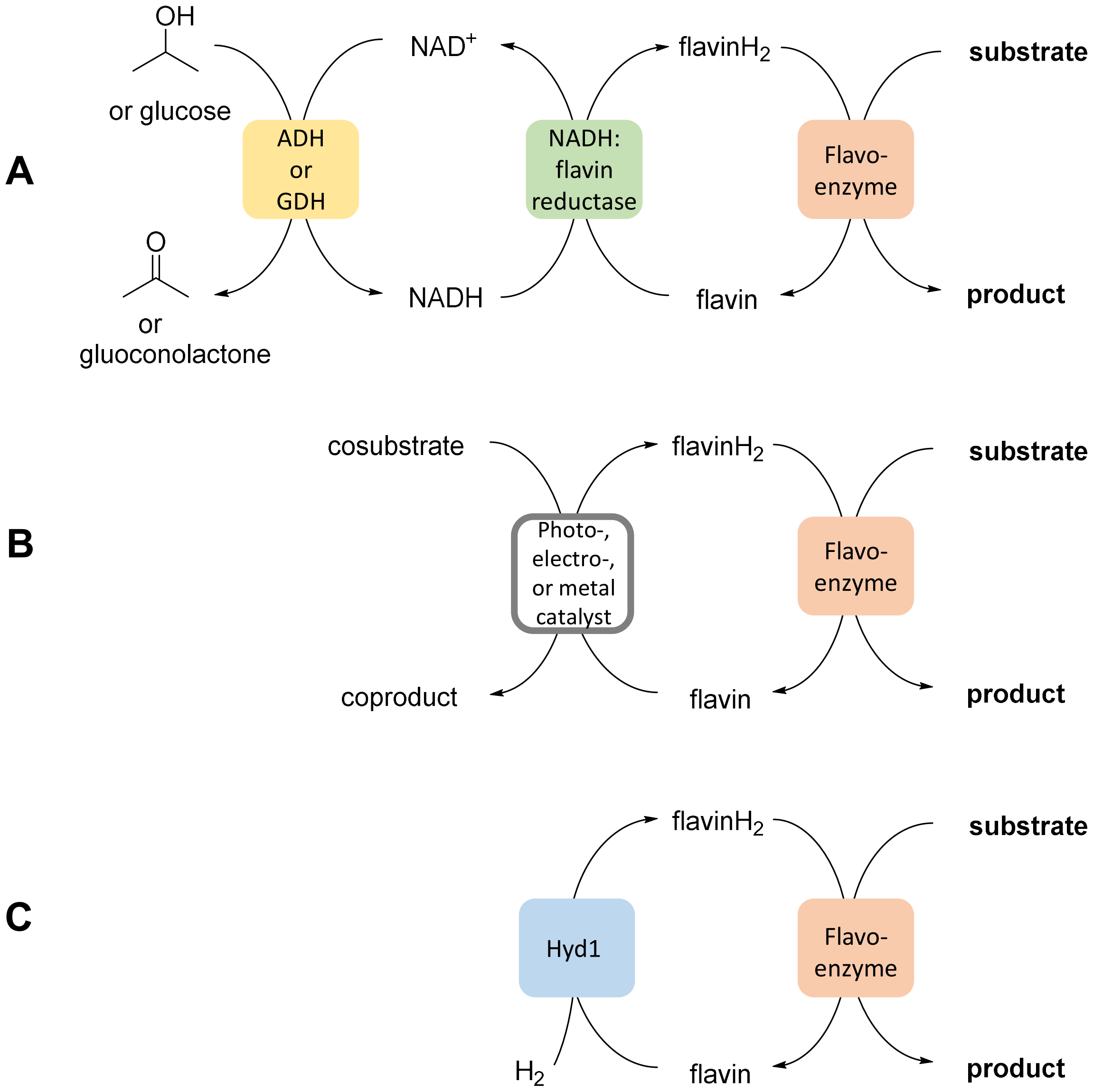

Figure S3. Current methods for regenerating flavin in situ. A. Current enzymatic flavin regeneration methods rely on NAD(P)H, which itself is continually regenerated using expensive, carbon-based sacrificial reductants. **B.** Other catalytic methods for flavin recycling tend to rely on cosubstrate additives. **C.** (This work) A simplified H_2-driven direct flavin reduction method using Hyd1 enzyme.

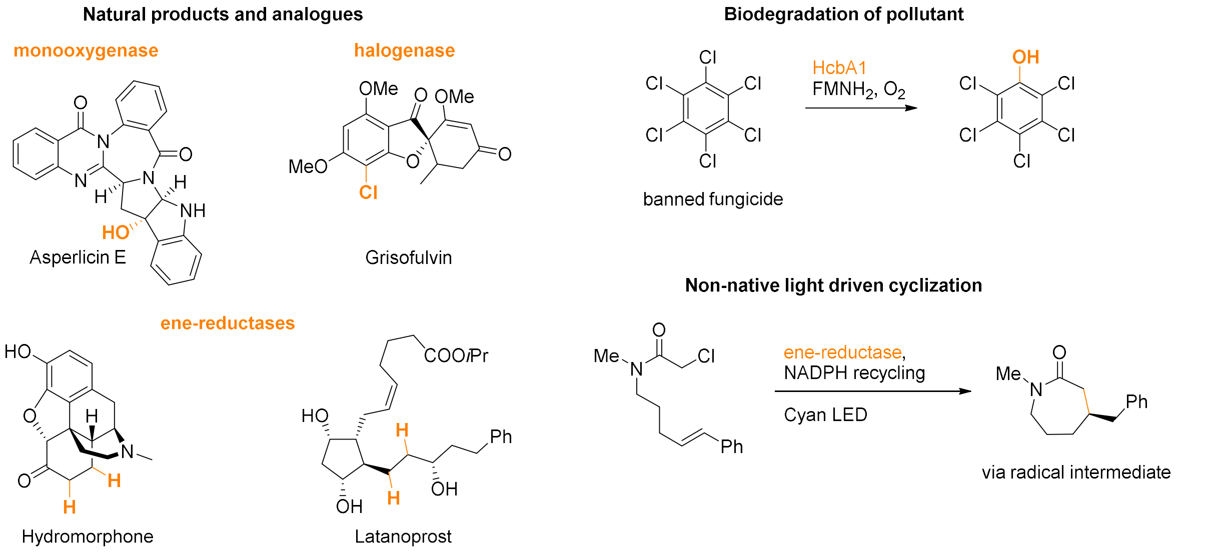

Figure S4. Current applications of flavin recycling. Examples of flavoenzymes applied toward natural products and analogues,[7–10] Degradation of an environmental pollutant,[11] and a non-native light-driven cyclisation.[12]

S4.1. Kinetic studies to determine the K_M (Michaelis-Menten constant) of Hyd1 for FAD

K_M (or apparent K_M) values are helpful in determining the concentration of flavin to use for the reactions. Absorbance values at 450 nm and 360 nm were too high to measure for the higher concentrations of FAD, but the absorbance at 512 nm could be used to calculate the concentration of FAD in accordance with Beer Lambert's Law. The calibration curve (**Figure S5 A)** provided an extinction coefficient of $\varepsilon = 460\ M^{-1}\ cm^{-1}$ for FAD at 512 nm. Using this, the initial activity of Hyd1 for varying concentrations of FAD was recorded. However, there was a decrease in enzyme activity at higher FAD concentrations (≥ 3.5 mM) suggestive of inhibition or other adverse effect on the enzyme. In order to determine an effective or apparent K_M for the enzyme functioning at low flavin concentrations, only the first five data points were fitted to a hyperbolic function to determine an apparent K_M at low flavin. **Figure S5 B** shows the Michaelis-Menten plot obtained for Hyd1 catalysed reduction of FAD under H_2. The data points corresponding to the blue circles and red squares each represent experiments performed on two different days under comparable conditions. The decrease in activity after 2.5 mM was observed during both the experiments, indicating a reproducible drop in activity at higher FAD concentrations.

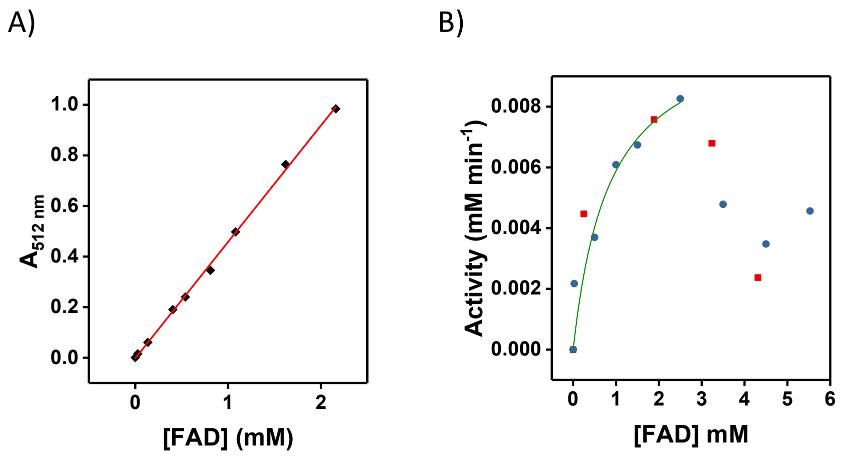

Figure S5. A. Calculating the extinction coefficient for FAD at 512 nm. **B.** Michaelis-Menten plot to determine an apparent $K_M = 0.9 \pm 0.5$ mM for Hyd1 catalysed reduction of FAD.

S4.2. Complete reduction of FMN to $FMNH_2$

To test stability over time, 57 µg Hyd1 was activated under H_2 at 22 °C for 58 h, then incubated in 0.08 mM FMN under H_2 (1 bar) in a sealed vessel for 62 h. Upon release of H_2, $FMNH_2$ partially oxidised under the N_2 atmosphere to 0.05 mM FMN (determined using UV-visible spectroscopy). The Hyd1 and FMN/$FMNH_2$ solution was placed back under H_2, and full reduction to $FMNH_2$ was noticed after 3.5 h (Figure S6), which demonstrates appreciable Hyd1 stability over 125 h (>5 days).

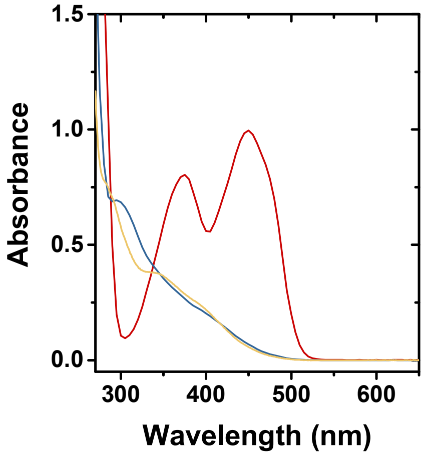

Figure S6. UV-visible spectra of FMN (red) and $FMNH_2$ produced by Hyd1 under H_2 (yellow) or sodium dithionite (blue). Reaction conditions for FMN reduction by Hyd1 (yellow): 800 µL scale, 0.08 mM FMN in Tris-HCl buffer (50 mM, pH 8, 25 °C), H_2 flow (cuvette head space), 57 µg Hyd1, 25 °C controlled by Peltier accessory. The full reduction of FMN by Hyd1 was completed during the experiment designed to test the stability of Hyd1 over time (>5 days). Reaction conditions for FMN reduction by sodium dithionite (blue): 800 µL scale, 0.08 mM FMN in Tris-HCl buffer (50 mM, pH 8, 25 °C), 0.15 mM sodium dithionite, 25 °C controlled by Peltier accessory. Control experiments to confirm role of Hyd1 and H_2 in flavin reduction.

S4.3. Control experiments for Hyd1 catalysed flavin reduction

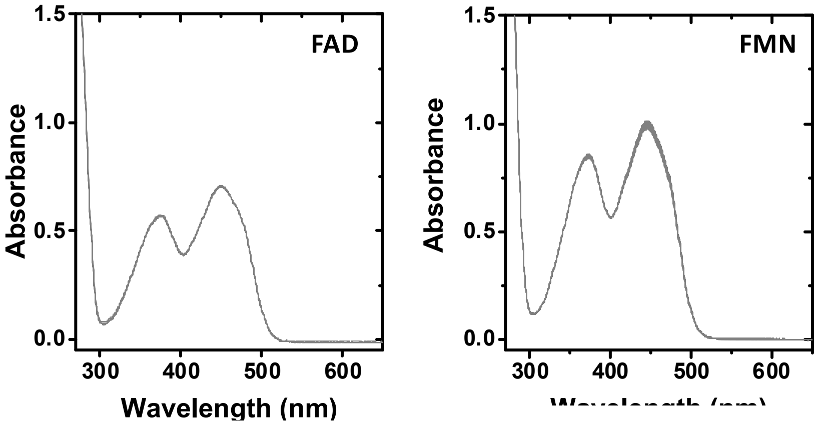

Figure S7. Background flavin reduction in absence of H_2

Reaction conditions: 800 µL scale, 0.1 mM flavin in Tris-HCl buffer (50 mM, pH 8, 25 °C), 40 µg Hyd1, 25 °C controlled by Peltier accessory. The Hyd1 specific activity for FAD and FMN reduction during this control reaction was 0.06 nmol min^{-1} mg^{-1} and 2.08 nmol min^{-1} mg^{-1} respectively.

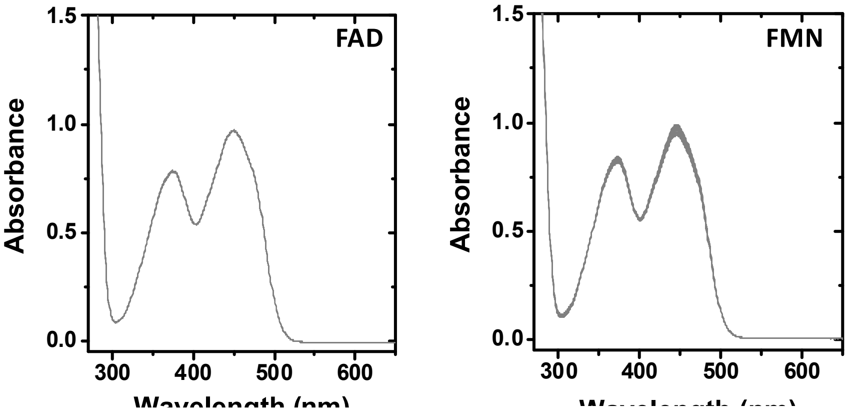

Figure S8. Background flavin reduction in absence of Hyd1
Reaction conditions: 800 µL scale, 0.1 mM flavin in Tris-HCl buffer (50 mM, pH 8, 25 °C), H_2 flow (cuvette head space), 25 °C controlled by Peltier accessory. The overall decrease in [FAD] and [FMN] amounts to 0.000 mM and 0.005 mM after 30 minutes respectively.

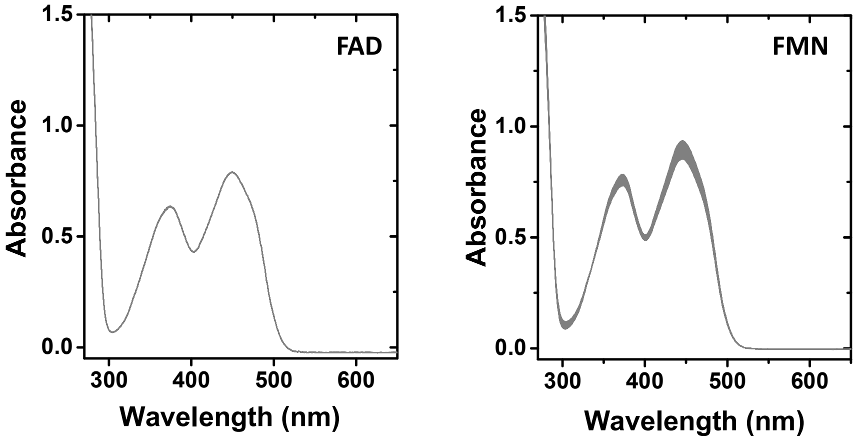

Figure S9. Background flavin reduction in the absence of H_2 and Hyd1
Reaction conditions: 800 µL scale, 0.1 mM flavin in Tris-HCl buffer (50 mM, pH 8, 25 °C), 25 °C controlled by Peltier accessory. The overall decrease in [FAD] and [FMN] amounts to 0.00 mM and 0.08 mM after 30 minutes respectively.

S4.4. Reduction of FMN using Hyd1 enzyme that contains or lacks the cytochrome

Experiments were conducted to determine whether the presence of the partner cytochrome, HyaC, impacts the flavin reduction activity. As shown in Figure S10 and Figure S11, the rate of FMN reduction was not affected significantly by the presence or absence of cytochrome. Hyd1 for these experiments was provided by Wangzhe Li and Sophie Kendall-Price, with advice from Dr Rhiannon Evans (University of Oxford). Hyd1 with cytochrome was separated by gel filtration. Hyd1 without cytochrome was prepared from a strain lacking the hyaC gene.

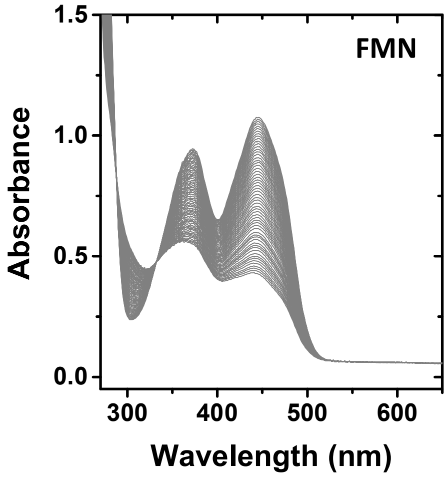

Figure S10. Flavin reduction in the presence of H_2 using a Hyd1 sample that does not contain the cytochrome. Reaction conditions: 800 µL scale, 0.1 mM FMN in Tris-HCl buffer (50 mM, pH 8, 25 °C), 40µg Hyd1 expressed without the cytochrome gene (activated for 16 h under H_2), 25 °C controlled by Peltier accessory. The Hyd1 specific activity observed for FMN for this reaction was 47.5 nmol min^{-1} mg^{-1}.

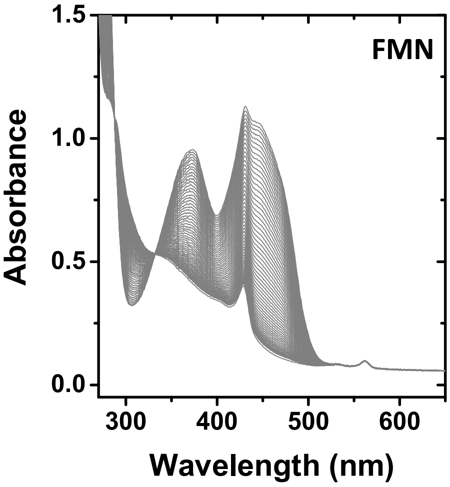

Figure S11. Flavin reduction under the presence of H_2 using Hyd1 containing the cytochrome. Reaction conditions: 800 µL scale, 0.1 mM FMN in Tris-HCl buffer (50 mM, pH 8, 25 °C), 60 µg Hyd1 containing the cytochrome subunit (activated for 17 h under H_2), 25 °C controlled by Peltier accessory. In this case, an absorbance change for oxidised vs reduced cytochrome is overlaid on the spectral changes from the FMN, giving rise to a sharp peak at around 425 nm.[13] The Hyd1 specific activity observed for FMN reduction in this reaction was 51.6 nmol min^{-1} mg^{-1}.

S4.5. Testing the stability of Hyd1-catalysed H_2-driven ketoisophorone reduction using *Ts*OYE under conditions designed to slow conversion rate

The reduction of **1** was performed following General Procedure B with 20 mM **1**, but using 71 µg Hyd1 that was activated under H_2 at 23 °C for 23 h. An additional 72 µg *Ts*OYE was added to the reaction mixture at 66 h which did not show any improvement in conversion rate as seen in the **Figure S12**. As the reaction approached full conversion, an additional 4.2 mM **1** was fed into the reaction mixture at 71 h. The reaction was stopped at 134 h and > 99% was observed.

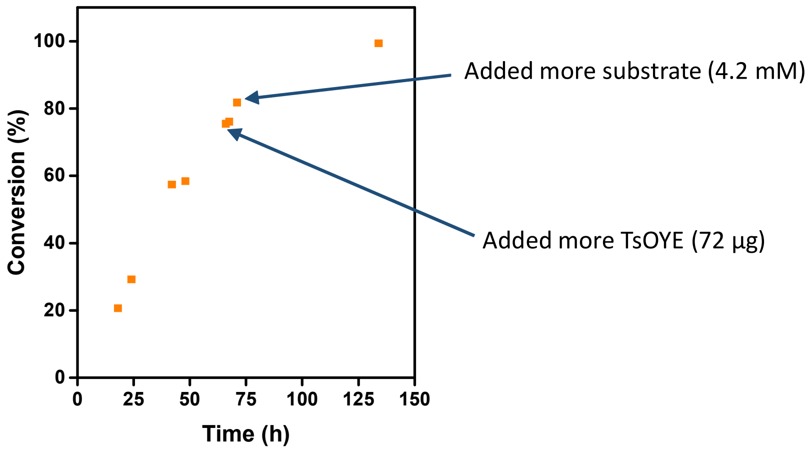

Figure S12. Catalytic stability of Hyd1 for FMN recycling in the conversion of ketoisophorone to (*R*)-levodione over 134 h.

S4.6. Reproducibility of the reactions

In order to prove the reproducibility of the reactions involving the flavin recycling system with *Ts*OYE for the reduction of ketoisophorone, three identical reactions were performed in separate reaction tubes and placed within the same pressure vessel. Conversions were calculated at 1 h and 3 h, standard deviation and error were calculated, and the results are compiled in **Table S1**.

Table S1. Results from a triplicate reaction to prove the reproducibility of the reaction

Entry	Conversion (%)	
	1 h	3 h
1	16.6	44.1
2	17.5	47.9
3	18.8	52.9
Standard deviation	1.1	4.4
Relative standard deviation	6.3	9.1

Reaction conditions: 300 µL scale, 0.1 mM FMN, 71 µg Hyd1, 72 µg *Ts*OYE, 10 mM ketoisophorone, Tris-HCl buffer (50 mM, pH 8.0, 25 °C), 1 vol% DMSO at room temperature (20–30 °C) in a pressure vessel (1 bar H_2).

S4.7. Control experiments for H_2-driven alkene reduction

Control experiments were performed to see if Hyd1 (entry 1) or ene-reductase (entry 2), alone, could lead to product formation. In addition, similar experiments were done in the absence of FMN (entry 3) or no enzyme (entry 4). The control experiments demonstrated the need for each reaction component for the reaction to be successful. The results of the experiment are shown below

Table S2. Control experiments for H_2-driven ketoisophorone (1) reduction

Entry	FMN	*Ts*OYE	Hyd1	Conversion to 2 (%)
1	✓	-	✓	0
2	✓	✓	-	0
3	-	✓	✓	0
4	✓	-	-	0

Reaction conditions: 600 µL scale, 0.1 mM FMN, 57 µg Hyd1, 72 µg *Ts*OYE , 10 mM **1**, Tris-HCl buffer (50 mM, pH 8.0, 25 °C), 1 vol% DMSO at room temperature (20–22°C) in a pressure vessel (1 bar H_2), 24 h.

Table S3. Control experiments for H_2-driven reduction of dimethyl itaconate (3)

Entry	FMN	Ene-103	Hyd1	Conversion to 4 (%)
1	✓	-	✓	0
2	✓	✓	-	0
3[a]	-	✓	✓	0
4	✓	-	-	0

Reaction conditions: 300 µL scale, 0.5 mM FMN, 71 µg Hyd1, ENE-103 (3 mg), 5 mM **3**, Tris-HCl buffer (50 mM, pH 8.0, 25 °C), 1 vol% DMSO at room temperature (20–26 °C) in pressure vessel (1 bar H_2), 18 h. [a]Reaction conditions: 600 µL scale, 142 µg Hyd1, ENE-103 (1.2 mg), 5 mM Dimethyl itaconate, Tris-HCl buffer (50 mM. pH 8.0, 25 °C), 1 vol% DMSO at room temperature (18–20 °C) in pressure vessel (1 bar H_2), 18 h.

Table S4. Control experiments for H_2-driven reduction of 4-Phenyl-3-buten-2-one (5)

Entry	FMN	Ene-107	Hyd1	Conversion to 6 (%)
1	✓	-	✓	0
2	✓	✓	-	2
3	-	✓	✓	6
4	✓	-	-	0

Reaction conditions: 300 µL scale, 0.5 mM FMN, 7.5 µL (0.003 U) Hyd1, ENE-107 (3 mg), 5 mM **5**, Tris-HCl buffer (50 mM, pH 8.0, 25 °C), 1 vol% DMSO at room temperature (20–26 °C) in pressure vessel (1 bar H_2), 18 h.

S4.8. Exemplary chiral-phase GC-FID spectra of H_2-driven alkene reductions

Reduction of alkene substrates were carried out and the reaction mixture was analysed by chiral GC-FID according to General Procedure C (see S3.3). Conversion to the corresponding products and the enantiomeric excess (%*ee*) were calculated based on the peak area of their respective peaks as shown in the figures below

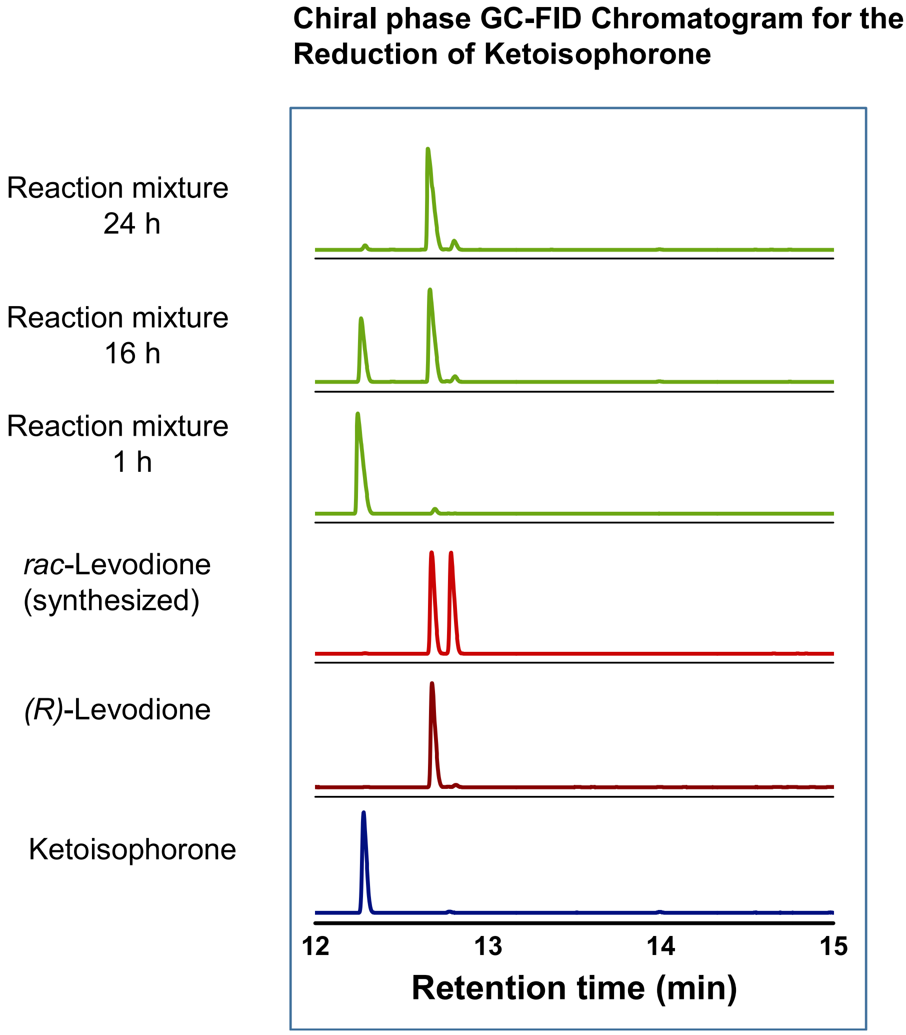

Figure S13. GC-FID results of ketoisophorone reductions (green). Ketoisophorone (purple, commercially available in ≥98% purity), (*rac*)-levodione (red, synthesised following literature procedure)[1] and (*R*)-levodione (burgundy, obtained from Baker's yeast fermentation) standards were diluted using EtOAc with 2 mM undecane as internal standard.

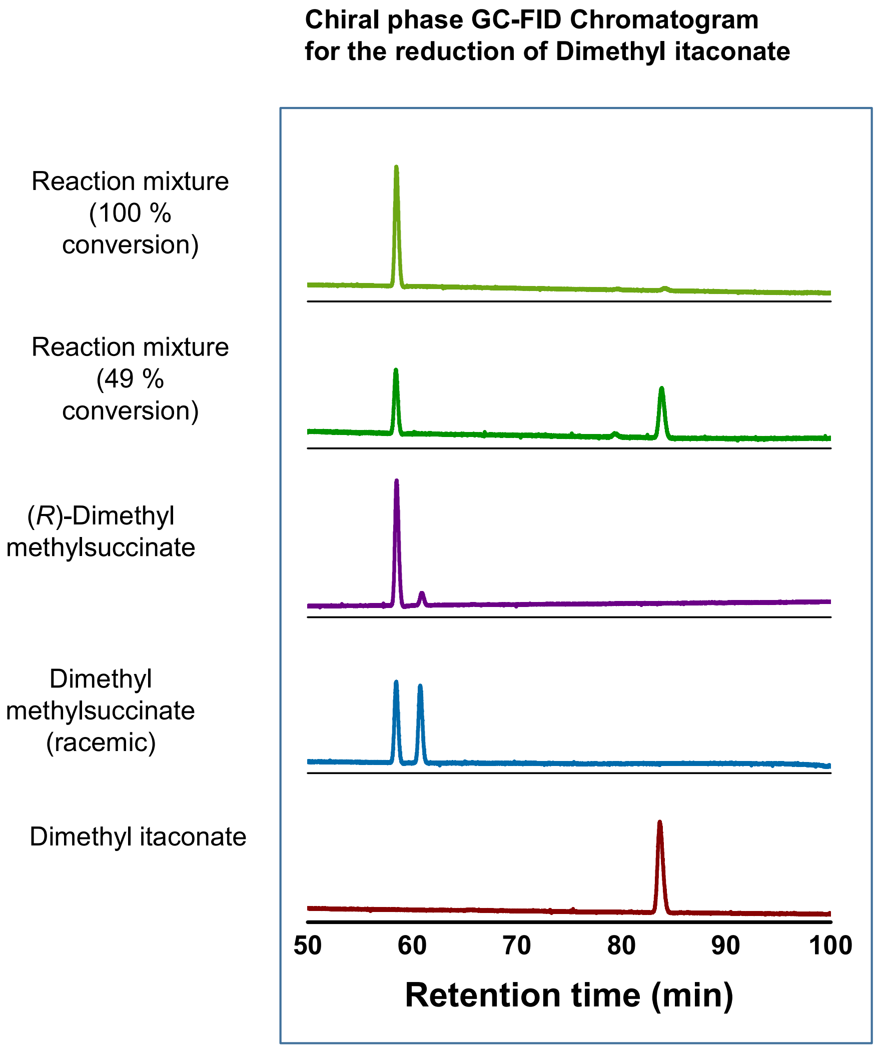

Figure S14. GC-FID results of dimethyl itaconate reductions (green). Dimethyl itaconate (maroon, commercially available, 98%), (*rac*)-dimethyl methyl succinate (blue, commercially available, 98%) and dimethyl (*R*)-methyl succinate (purple, commercially available, 99%) standards were diluted using EtOAc.

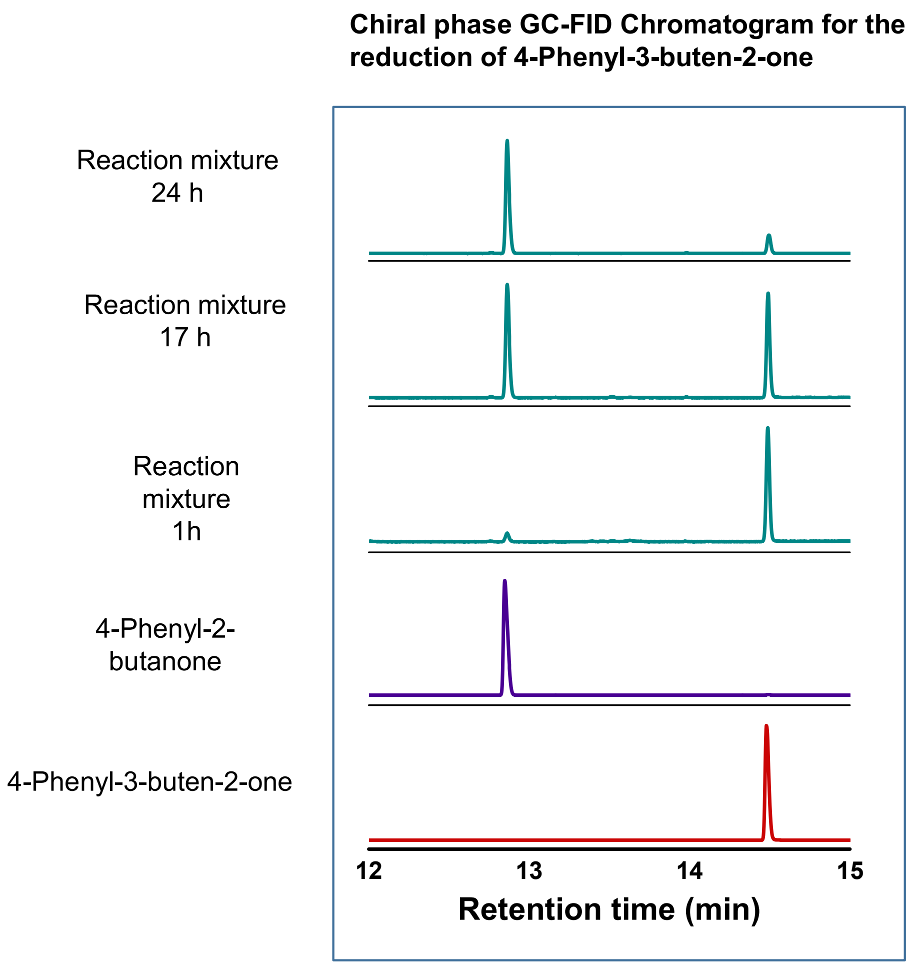

Figure S15. GC-FID results of 4-phenyl-3-buten-2-one reductions (blue green). 4-phenyl-3-buten-2-one (red, commercially available, 99%) and 4-phenyl-2-butanone (violet, commercially available, 98%) standards were diluted using EtOAc.

S5. References

[1] S. Mathew, M. Trajkovic, H. Kumar, Q.-T. Nguyen, M. W. Fraaije, *Chem. Commun.* **2018**, *54*, 11208–11211.

[2] H. A. Reeve, L. Lauterbach, O. Lenz, K. A. Vincent, *ChemCatChem* **2015**, *7*, 3480–3487.

[3] D. Johannes Opperman, L. Ann Piater, E. van Heerden, *J. Bacteriol.* **2008**, *190*, 3076–3082.

[4] D. J. Opperman, B. T. Sewell, D. Litthauer, M. N. Isupov, J. A. Littlechild, E. van Heerden, *Biochem. Biophys. Res. Commun.* **2010**, *393*, 426–431.

[5] P. Macheroux, in *Methods Mol. Biol.* (Eds.: S.K. Chapman, G.A. Reid), Humana Press, Totowa, NJ, **1999**, pp. 1–7.

[6] M. C. R. Rauch, M. Pesic, M. M. E. Huijbers, M. Pabst, C. E. Paul, M. Pešić, I. W. C. E. Arends, F. Hollmann, *BBA- Proteins Proteom.* **2020**, *1868*, 140303.

[7] R. A. Cacho, Y. H. Chooi, H. Zhou, Y. Tang, *ACS Chem. Biol.* **2013**, *8*, 2322–2330.

[8] M. L. Contente, P. Zambelli, S. Galafassi, L. Tamborini, A. Pinto, P. Conti, F. Molinari, D. Romano, *J. Mol. Catal. B.-Enzym.* **2015**, *114*, 7–12.

[9] S. W. Haynes, X. Gao, Y. Tang, C. T. Walsh, *J. Am. Chem. Soc.* **2012**, *134*, 17444–17447.

[10] B. Boonstra, D. A. Rathbone, N. C. Bruce, *Biomol. Eng.* **2001**, *18*, 41–47.

[11] S. Adak, T. P. Begley, *Biochemistry* **2019**, *58*, 1181–1183.

[12] K. F. Biegasiewicz, S. J. Cooper, X. Gao, D. G. Oblinsky, J. H. Kim, S. E. Garfinkle, L. A. Joyce, B. A. Sandoval, G. D. Scholes, T. K. Hyster, *Science* **2019**, *364*, 1166–1169.

[13] V. Rodríguez-Roldán, J. M. García-Heredia, J. A. Navarro, M. Hervás, B. De la Cerda, F. P. Molina-Heredia, M. A. De la Rosa, *Biochem. Biophys. Res. Commun.* **2006**, *346*, 1108–1113.
